# Sa12b Improves Biological Activity of Human Degenerative Nucleus Pulposus Mesenchymal Stem Cells in a Severe Acid Environment by Inhibiting Acid-Sensitive Ion Channels

**DOI:** 10.3389/fbioe.2022.816362

**Published:** 2022-01-28

**Authors:** Ziyu Wang, Letian Han, Haoyu Chen, Shengquan Zhang, Sumei Zhang, Hua Zhang, Yuhao Li, Hui Tao, Jie Li

**Affiliations:** ^1^ Department of Clinical Laboratory, The First Affiliated Hospital of Anhui Medical University, Hefei, China; ^2^ Department of Orthopedics and Spine Surgery, The First Affiliated Hospital of Anhui Medical University, Hefei, China; ^3^ Department of Biochemistry and Molecular Biology, School of Basic Medical Sciences, Anhui Medical University, Hefei, China

**Keywords:** nucleus pulposus mesenchymal stem cells, acid-sensitive ion channels, Sa12b, acidsensitive ion channel inhibitors, intervertebral disc degeneration

## Abstract

Sa12b is a wasp peptide that can inhibit acid-sensitive ion channels (ASICs). The biological effects of nucleus pulposus mesenchymal stem cells (NP-MSCs) have not been investigated. Therefore, this study investigated the effect of Sa12b on the biological activity of NP-MSCs through ASICs in the acidic environment of intervertebral disc degeneration (IVDD). In this study, NP-MSCs were isolated from the nucleus pulposus (NP) in patients who underwent lumbar disc herniation surgery, identified by flow cytometry and tertiary differentiation, and cultured *in vitro* in an acidic environment model of IVDD with a pH of 6.2. Proliferation, and apoptosis were observed after different Sa12b concentrations were added to P2 generation NP-MSCs. The Ca^2+^ influx was detected using flow cytometry and laser confocal scanning microscopy, and qPCR was used to detect the relative expression of stem cell–associated genes (Oct4, Nanog, Jag1, and Notch1), the relative expression of extracellular matrix (ECM)–associated genes (collagen II, aggrecan, and SOX-9), and the relative expression of genes encoding ASICs (ASIC1, ASIC2, ASIC3, and ASIC4). Western blotting was used to detect the protein expression of collagen II and aggrecan in different treatment groups. Cells isolated and cultured from normal NP were spindle-shaped and adherent, and they exhibited expansion *in vitro*. Flow cytometry results showed that the cells exhibited high expression of CD73 (98.1%), CD90 (97.5%), and CD105 (98.3%) and low expression of HLA-DR (0.93%), CD34 (2.63%), and CD45 (0.33%). The cells differentiated into osteoblasts, adipocytes, and chondrocytes. According to the International Society for Cellular Therapy criteria, the isolated and cultured cells were NP-MSCs. With an increase in Sa12b concentration, the cell proliferation rate of NP-MSCs increased, and the apoptosis rate decreased significantly, reaching the optimal level when the concentration of Sa12b was 8 μg/μl. When the Sa12b concentration was 8 μg/μl and contained the ASIC non-specific inhibitor amiloride, the Ca^2+^ influx was the lowest, followed by that when the Sa12b concentration was 8 μg/μl. The Ca^2+^ influx was the highest in the untreated control group. qPCR results showed that as the concentration of Sa12b increased, the relative expression of Oct4, Nanog, Jag1, Notch1, collagen II, aggrecan, and SOX-9 increased, while that of ASIC1, ASIC2, ASIC3, and ASIC4 decreased. The difference was statistically significant (*p* < 0.05). In conclusion, Sa12b can improve the biological activity of NP-MSCs in severely acidic environments of the intervertebral disc by reducing Ca^2+^ influx *via* AISC inhibition and, probably, the Notch signaling pathway. This study provides a new approach for the biological treatment of IVDD. Inhibition of AISCs by Sa12b may delay IVDD and improve low back pain.

## Introduction

Low back pain (LBP) is one of the most common public health problems and is the leading cause of disability worldwide. It causes a severe social burden and mainly occurs among adults 25–49 years of age (Dieleman et al., 2020; O'Sullivan et al., 2020). Current treatments for LBP include mainly conservative management and surgery, which cannot cure the fundamental causes (Corp et al., 2021). According to previous reports, intervertebral disc (IVD) degeneration (IVDD) is the leading cause of LBP. In addition to standard conservative management and surgical treatment, experimental cell regeneration technology has also received extensive attention from researchers as a treatment strategy for lumbar IVDD (Kos et al., 2019).

The IVD is an avascular and non-renewable tissue composed of the central nucleus pulposus (NP), peripheral fibrous annulus, and upper and lower cartilage endplates. The NP plays a central role in IVD function and consists of nucleus pulposus cells (NPCs) and extracellular matrix (ECM) components. After the degeneration of the NP, nutrient metabolism is unbalanced, and the secretion of ECM components decreases, especially that of collagen II and aggrecan. This reduces the water content in the NP, increases the osmotic pressure, decreases the pH value, induces hypoxia, and accelerates internal disc disruption (IDD). In addition, the drop in disc height eventually leads to IVDD. Therefore, regulation of NPC function and restoration of the ECM metabolic balance is necessary to delay IVDD progression (Zhang et al., 2016). Recently, several studies have shown that nucleus pulposus mesenchymal stem cells (NP-MSCs) can be regenerated and differentiated into NP-like cells in IVDD. Biological treatments, including those based on cytokines, stem cells, and other biological materials, can promote the expression of proteoglycans in the degenerated NP, increase the water content in the IVD, and thus play a role in delaying IVDD (Tao et al., 2014; Tao et al., 2015; Bowles and Setton, 2017).

Relevant studies have shown that with IVDD, many changes occur in the internal environment of IDD, resulting in hypoxia, low pH, hypertonicity, and low nutrition (Colombier et al., 2014). A low pH can reduce the biological activity of NP-MSCs in IVDD. In addition, the change in pH *in vivo* is mainly controlled by acid-sensitive ion channels (ASICs) to regulate the biological activity of cells (Vullo and Kellenberger, 2020). ASICs are gated ion channels that belong to an essential member of the epithelial sodium channel/degenerin (DEG/ENaC) family of sodium-selective channels. They are important receptors for changes in the acidic environment *in vivo* and are widely distributed in various body tissues. ASICs are encoded by four genes and consist of seven subunits: ASIC1a, ASIC1b, ASIClb2, ASIC2a, ASIC2b, ASIC3, and ASIC4, which are expressed in both normal and degenerative NP and are positively correlated with the degree of degeneration (Cuesta et al., 2014). In addition, studies have found that changes in the acid environment of IDD regulate the biological activity of NP-MSCs through ASICs. Human degenerative NP-MSCs express ASIC1, ASIC2, ASIC3, and ASIC4, and this expression is inversely proportional to pH (Liu et al., 2017).

Carmen Hernández et al. purified and extracted the short peptide Sa12b (EDVDHVFLRF) from the venom of the solitary wasp Sphex argentatus and found that it belonged to a new type of non-specific ASIC inhibitor. Preincubation with Sa12b reversibly inhibited the amplitude of the ASIC current peak (IC50∼81 nM) in rat DRG neurons in a concentration-dependent manner but had no consistent effect on the time course of desensitization or the persistent component of the current. Thus, the inhibitory effect of Sa12b (*IC50* = 81 nM) on ASIC current is equivalent to that caused by peptides of plant and animal origin, such as chlorogenic acid, gastrodin, phenol, APETx2, and mambalgines (Hernández et al., 2019). In addition, related studies have shown that when the pH value of the *in vivo* environment changes, the opening probability, ion permeability, and ion selectivity of ASICs can be used to adjust the amount of Ca^2+^ transported into cells, thereby adjusting the biological activity of cells (Zhou et al., 2016). However, it is unclear whether Sa12b mediates Ca^2+^ influx through ASICs, thereby affecting the biological activity of NP-MSCs and alleviating or inhibiting IVDD. Thus, this study aimed to investigate the effect of Sa12b on the biological activity of NP-MSCs through ASICs in the acidic environment of intervertebral disc degeneration (IVDD).

## Materials and Methods

### Preparation of Sa12b Mother Liquor

The Sa12b mother liquor used in this study was custom-synthesized at Sangon Biotech (purity >90%, Shanghai, China). The peptide sequences are presented in Table 1. This peptide powder was dissolved in phosphate buffered saline (PBS; Gibco) at a final concentration of 1% (w/v, 10 mg/ml) and sonicated on ice for 5 min. The peptide solution was filter-sterilized using syringe-driven filter units (0.22 μm HT Tuffryn membrane, Pall Corp., Ann Arbor, MI, United States) prior to use.

**TABLE 1:** The peptide sequences of Sa12b.

name	Sequence
Sa12b	EDVDHVFLRF-

### Isolation and Culture of Cells

Isolation of cells from patients with lumbar disc herniation was performed; Table 2 provides detailed information about the age, sex, and disease status of the subjects. In accordance with the Declaration of Helsinki, all procedures for the study were approved by the local ethics committees of our institution and were performed with the informed consent of the patients. After washing the sample twice with PBS (Gibco), the annulus fibrosus and cartilaginous endplate were carefully removed, and the NP tissue was cut into approximately 1 mm^3^ sections and digested in 5% CO_2_ at 37°C with collagenase II solution (0.02 mg/ml). After 8 h, the cells were precipitated by centrifugation at 1,500 rpm for 5 min. The supernatant was removed, and the free cells and partially digested tissue were cultured as explants in a standard MSC expansion medium consisting of Dulbecco’s modified Eagle’s medium–low glucose (Hyclone, United States), 10% fetal bovine serum (FBS; Gibco, United States), and penicillin/streptomycin (United States, Gibco) in a humidified incubator at 37°C and 5% CO_2_. After 24 h, suspended cells and debris were removed from the medium, and the medium was completely replaced every 3–4 days to promote adherent cell growth. When the cells reached approximately 90% confluence, the culture was passaged at a ratio of 1:3. The second-generation cells were harvested using trypsin-EDTA solution (United States, Gibco) and tested.

**TABLE 2:** Sample information.

Case NO.	Age (years)	Gender	Pfirrmann grading
Case 1	32	female	Ⅴ
Case 2	26	male	Ⅴ
Case 3	23	male	Ⅳ
Case 4	26	female	Ⅳ

### Identification of NP-MSC

After digesting NP-MSCs with 0.25% trypsin (Biosharp, United States), the cells were washed and resuspended in 100 µl PBS (Sigma, United States) containing 1% FBS. Each tube contained 5 µl of the following antibodies, according to the recommendations of the International Society for Cell Therapy: CD34-APC, CD73-FTTC, CD45-PE, CD90-FTTC, CD105-PE, and HLA-DR-APC (eBioscience, United States). In each case, isotype control (eBioscience, United States) was used. After incubation with the antibody for 30 min at room temperature, the cells were washed with PBS, and the supernatant was discarded. The cells were resuspended in 200 ul of PBS (Sigma, United States) and analyzed using a flow cytometer (BD, United States). Immunophenotyping analysis was performed to identify the percentage of positive cells and the fluorescence intensity.

### Osteogenic Differentiation

The normal medium was replaced with an osteogenic differentiation medium (Cyagen, United States). The differentiation process lasted for 14 days, and the medium was changed every 2–3 days. After differentiation, the medium was removed, and the cells were washed with PBS and fixed with 4% paraformaldehyde solution (Sangon Biotech, China) at room temperature for 30 min. After washing twice with PBS, the cells were stained with alizarin red working solution (Cyagen, United States) for 15 min. Finally, the culture was washed three times with PBS and observed under an optical microscope.

### Adipogenic Differentiation

NP-MSCs from the second passage were seeded in a six-well plate at a density of 5 × 104 cells/cm^2^. When the cells reached 100% confluence, the normal medium was replaced with adipogenic differentiation medium A (Cyagen, United States). After 3 days, differentiation medium A was replaced with adipogenic differentiation medium B (Cyagen, United States) for 24 h. Then, medium B was replaced with medium A again. This cycle was repeated for three to four cycles for a total of 28 days. After differentiation, the medium was removed from the wells, and the cells were washed with PBS and fixed with 4% paraformaldehyde solution for 30 min. After washing twice with PBS, the cells were stained with Oil Red O working solution (Cyagen, United States) for 30 min at room temperature. The culture was then washed three times with PBS and observed under an optical microscope.

### Chondrogenic Differentiation

NP-MSCs from the second passage were resuspended in chondrogenic differentiation basal medium (Cyagen, United States), centrifuged at 1,500 rpm for 5 min, and the supernatant was discarded. The cells were resuspended in a complete chondrogenic differentiation medium (Cyagen, United States) at a density of 5 × 105 cells/ml. Next, 500 μl of the cell suspension was placed in a 15 ml centrifuge tube containing 2.5 × 105 cells and centrifuged at 1,500 rpm for 5 min to form a pellet. The pellet was cultured at 37°C in 5% CO_2_ in a complete chondrogenic differentiation medium without interference. After 3 days, the medium was changed, and the bottom of the test tube was flicked to ensure that the cartilage ball floated freely. After that, the differentiation medium was changed every 2 days, and the differentiation process lasted for 28 days. When the diameter of the cartilage ball increased to 3 mm, tissue sections were fixed in formalin and embedded in paraffin (Sangon Biotech, China). Finally, the sections were deparaffinized and hydrated with distilled water. The sections were then stained with Alcian Blue (Cyagen, United States) for 30 min. They were washed with running tap water for 2 min and then with distilled water several times. Finally, the sections were observed under an optical microscope.

### Preparation of Media With Different pH Values

Media with different pH values were prepared by adding an appropriate amount of sterilized HCl (1 mol/L) and NaOH (1 mol/L) to the culture medium and monitoring the pH values using a pH microelectrode (Ramagnetic phs-25, China). After pH values (7.4, 6.2) were obtained, the medium was kept in 5% CO_2_ at 37°C for 3 days to establish a pH balance (CO_2_-dependent).

### Cell Proliferation Assay

The second-generation NP-MSCs were inoculated in 96-well plates (200 μl medium per well) at a density of 3 × 104 cells/ml. These cells were cultured in a complete medium with a pH of 6.2 at 37°C with 5% CO_2_. To observe the effect of Sa12b on proliferation, the experimental group also contained 20, 40, 60, and 80 µg Sa12b. Sub-samples were collected from three wells on days 1, 3, 5, 7, and 9, and 20 µl of CCK-8 (Japan, DOJINDO) was added to the cells, which were then incubated for 2 h. The isotype group did not contain any cells. The absorbance of each group was measured using a Spectrum MAX microplate reader.

### Cell Cytotoxicity Assessment

NP-MSCs from the second passage were seeded in a 12-well plate at a density of 1 × 105 cells/ml. These cells were cultured for 2 days at different pH levels at 37°C and 5% CO_2_. CAM and the nucleic acid dye propidium iodide (PI, Sigma, United States) were used to label live and dead NP-MSCs, respectively. Briefly, the cells were incubated with 2 μmol/L CAM and 5 μmol/L PI at room temperature for 30 min in the dark and then gently rinsed with PBS three times. A fluorescence microscope was used to collect the images.

### Apoptosis Measurement of NP-MSCs

NP-MSCs from the second passage were seeded in a 12-well plate at a density of 1 × 105 cells/ml. These cells were cultured for 2 days at different pH levels at 37°C and 5% CO_2_. To observe the effect of Sa12b on cell apoptosis, the experimental group also contained Sa12b at various concentrations (2, 4, 6, and 8 μg/μl), with four replicate wells in each group. The adherent cells were collected by trypsinization without EDTA (Biosharp, United States) and washed twice with PBS. In addition, Annexin V-FITC (5 µL) and PI (KeyKey BioTECH, China) were added to each group and incubated at room temperature in the dark for 15–20 min, and flow cytometry (United States, BD) was used within 1 h to detect the percentage of apoptotic cells.

### Quantitative Real-Time PCR Analysis of Gene Expression

NP-MSCs from the second passage were seeded in T-25 culture flasks at a density of 5 × 105 cells/ml and cultured in 5% CO_2_ at 37°C at different pH levels for 7, 14, and 28 days. To observe the effect of Sa12b on cell apoptosis, the experimental group also contained Sa12b at various concentrations (2, 4, 6, and 8 μg/μl). TRIzol (Ambion, United States) was used to extract total RNA according to the manufacturer’s instructions, and the RNA samples were treated with DNase/RNase-free water. A Nanodrop ND-1000 spectrophotometer (Nanodrop Technologies, United States) was used to determine the quality and quantity of RNA. A reverse transcription reagent (Takara, Japan) was used to obtain cDNA from total RNA. A total of 1,000 ng of RNA was mixed with 2 µl of 5× PrimeScript RT^®^MasterMix and RNase-free ddH2O (the total system volume was 10 µl). The mixed solution was first incubated at 37°C for 15 min and then at 85°C for 5 s, and stored at −80°C for qPCR. All genes were analyzed by qPCR, and GAPDH was used as a control. SYBR Premix Ex Taq PCR kit (Takara, Japan) and LightCycler (Roche, Switzerland) were used for qPCR analysis. Premier software (version 5.0) was used to design the primers for all genes, as shown in Table 3.

**TABLE 3:** Primers used in qPCR.

Genes	Sense primer	Antisense primer
DAPDH	GAAGGTCGGAGTCAACGG	GGA​AGA​TGG​TGA​TGG​GAT​T
ASIC1	TTC​AAG​GTG​GTC​TTC​ACA​CGC​TAT​G	AGG​TAC​TCG​TCC​TGC​TGG​ATG​TC
ASIC2	TCC​TAC​TAC​TTC​TCC​TAC​CAG​CAT​GTC	CGG​AAG​CCA​TTC​AGG​TTA​CAG​AGG
ASIC3	GCC​TGA​GAA​CTT​CAC​CAC​GAT​CTT​C	GCA​CGT​CCA​GCA​TGA​TGT​CCA​G
ASIC4	ACC​ATC​TGC​CCA​CCA​AAT​ATC​TAC​ATC	CTC​TTT​CCC​ATA​GCG​TGT​CAG​GTT​G
Aggrecan	ACG​GCT​TCT​GGA​GAC​AGG​ACT​G	CTG​GGA​TGC​TGG​TGC​TGA​TGA​C
SOX-9	AGG​AGA​GCG​AGG​AGG​ACA​AGT​TC	TGT​TCT​TGC​TGG​AGC​CGT​TGA​C
Collagen II	GGA​GCA​GCA​AGA​GCA​AGG​AGA​AG	TCA​TCT​GGA​CGT​TGG​CAG​TGT​TG
Notch1	GCC​TCA​ACA​TCC​CCT​ACA​AG	CAC​GAA​GAA​CAG​AAG​CAC​AAA
Oct4	ACA​CTG​CAG​CAG​ATC​AGC​CAC	CCA​GAG​GAA​AGG​ACA​CTG​GTC
Nanog	GCT​TTG​AAG​CAT​CCG​ACT​GT	TTT​GGG​ACT​GGT​GGA​AGA​AT
Jagged	CGA​GGA​CTA​TGA​GGG​CAA​GA	CTT​CAG​GTG​TGT​CGT​TGG​AA

### Western Blot Analysis

NP-MSCs from the second passage were seeded in a 6-well plate at a density of 1 × 105 cells/ml. These cells were cultured in 5% CO_2_ at 37°C at different pH levels for 14 days. To observe the effect of Sa12b on cell apoptosis, the experimental group also contained Sa12b at various concentrations (2, 4, 6, and 8 μg/μl). The cells were washed three times with ice-cold PBS, and total protein was extracted using RIPA buffer containing 1% PMSF. Protein concentration was measured using a BCA protein quantification kit (Takara, Japan). The protein was electrophoresed by 10% sodium dodecyl sulfate-polyacrylamide gel electrophoresis (SDS-PAGE) and then transferred to a polyvinylidene fluoride (PVDF) membrane (Millipore, Massachusetts, United States). After blocking with 5% skimmed milk in Tris-buffered saline containing 0.1% Tween-20 (TBST) at room temperature for 1 h, the membrane in TBST was incubated with appropriate primary antibodies: anti-aggrecan antibody, anti-collagen II antibody (maintained at 4°C overnight), and anti-GAPDH antibody (1:1,000; Santa Cruz). The membrane was then incubated with horseradish peroxidase (HRP)-labeled secondary immunoglobulin G (1:1,000, Santa Cruz) at room temperature for 1 h. After washing the membrane three times with TBST, the immunoreactivity was detected with an enhanced chemiluminescence (ECL, Millipore) substrate, and the optical density was measured using Quantum One software (BioRad Laboratories Inc., Munich, Germany). GAPDH was used as the loading control.

### Calcium Imaging

NP-MSCs from the second passage were seeded at a density of 1 × 105 cells/ml in a special cell culture dish for confocal microscopy and cultured in an acidic environment (*pH* = 6.2) in 5% CO_2_ at 37°C. The cells were then washed three times with D-Hanks’ solution and incubated with 5 μM Fluo-3-AM (Dojindo Laboratories, Japan) for 30 min at 37°C, followed by three washes and an additional incubation in normal Hanks’ solution for 15 min. Fluo-3-AM was excited at 488 nm using a laser scanning confocal microscope, and the emission was measured at 510 nm.

### Detection of [Ca^2+^] i Using Flow Cytometry

Cell suspensions in Eppendorf tubes were washed three times with D-Hanks’ solution and incubated with 5 μM Fluo-3-AM (Dojindo Laboratories, Japan) for 30 min at 37°C. Then, the cells were washed three times for 5 min and incubated in normal Hanks’ solution for 15 min. Fluo-3-AM was excited at 488 nm using a flow cytometer, and emission was measured at 510 nm.

### Statistical Analysis

All data were statistically analyzed using SPSS 23.0 and are presented as the mean ± standard deviation. ANOVA of the factorial design was performed to analyze the main effect and the interaction between the groups and the time periods, and one-way ANOVA was performed for multiple-group comparisons. The Student-Newman-Keuls test (homogeneity of variance) or Tamhane’s test (heterogeneity of variance) was performed to compare any two groups. Statistical significance was set at *p* < 0.05.

## Results

### The Experimental Cells Were Identified as NP-MSCs Through Immunophenotyping and *In Vitro* Pluripotent Differentiation

**FIGURE 1:**
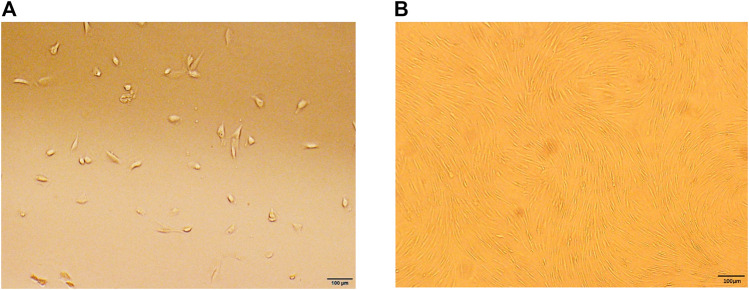
Characteristics of cell culture *in vitro*. **(A)** Human degenerative NP-MSCs exhibited a short, rod-like shape in primary culture. **(B)** Human degenerative NP-MSCs showed uniform spindle filament spiral growth after subculture.

**FIGURE 2:**
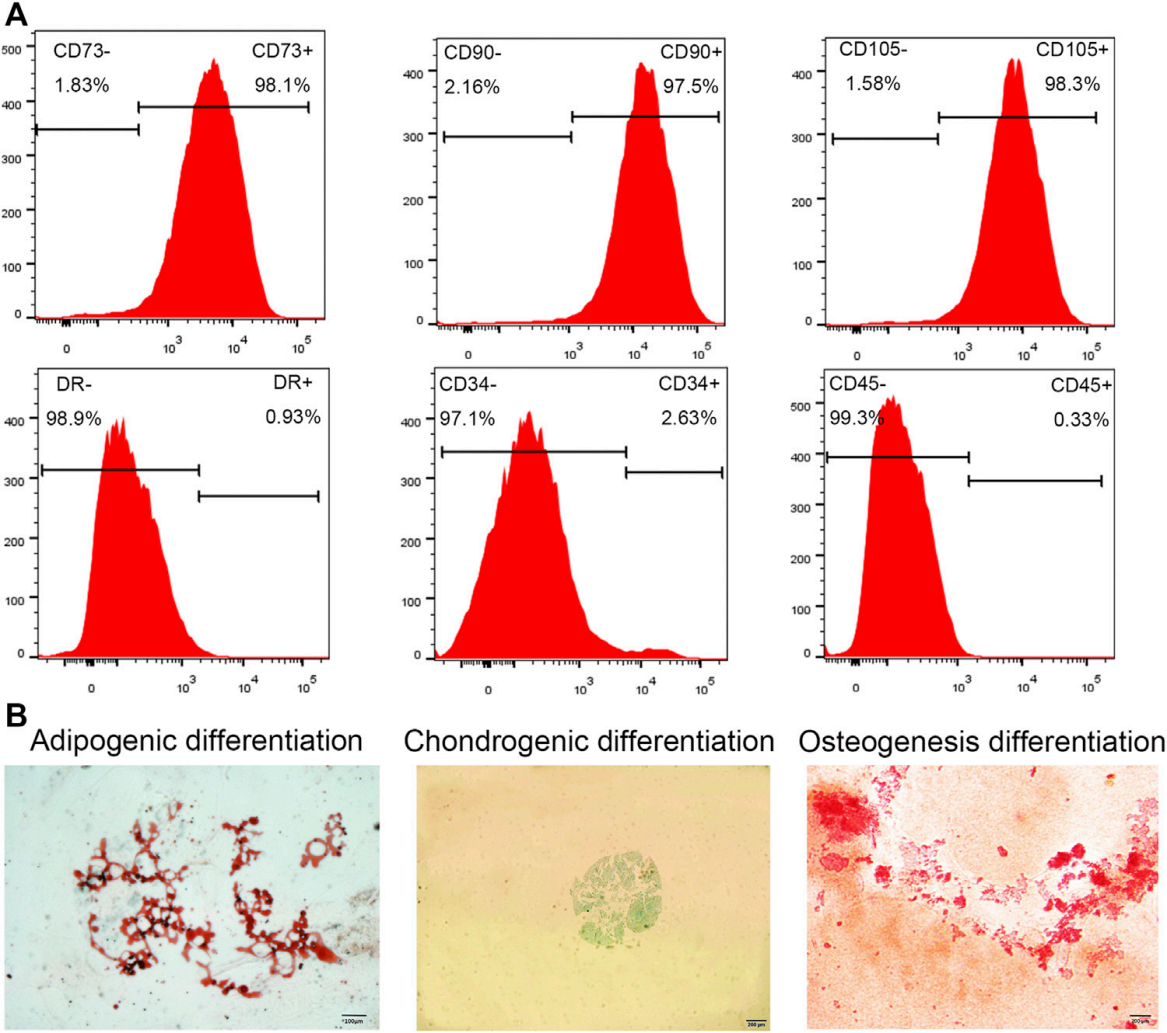
Cell phenotype identification and cell differentiation. **(A)** Flow cytometry analysis showed high expression of the cell surface markers CD105, CD90, and CD73, while CD45, CD34, and HLA-DR expression was absent. **(B)** Three lineage differentiation of NP-MSCs. Oil red O staining of NP-MSC intracellular lipid vacuoles indicated adipogenic differentiation; Alcian blue staining of cartilage indicated chondrogenic differentiation; Alizarin red staining of calcium nodules indicated osteogenic differentiation.

The cells were extracted from human IVDs, isolated, and cultured *in vitro*. The primary cells showed a short spindle shape after 3–5 days (Figure 1A). After approximately 2 weeks of passage, the growth rate of the primary cells was significantly faster than that of the primary cells, with spiral growth, uniform morphology, and a characteristic spindle shape (Figure 1B). Flow cytometry showed that the cultured cells had high expression of CD73, CD90, and CD105 and low expression of CD34, CD45, and HLA-DR (Figure 2A). In addition, after the cells were incubated in differentiation induction culture, strongly stained mineralized nodules indicated osteogenic differentiation, oil red O staining of intracellular lipid vacuoles indicated adipogenic differentiation, and staining with sulfated proteoglycans indicated chondrogenic differentiation (Figure 2B). In summary, the cells met the evaluation criteria of the International Society for Cellular Therapy (ISCT) for NP-MSCs.

### Sa12b Alleviated Proliferation Inhibition of NP-MSCs in a Severely Acidic Environment

As shown in Figure 2A, compared with the normal culture environment (*pH* = 7.4), the proliferation of human degenerative NP-MSCs cultured in an acidic environment (*pH* = 6.2) was significantly inhibited (*p* < 0.001); however, after adding different concentrations of Sa12b (0, 2, 4, 6, 8 μg/μl) to the environment, the proliferation inhibition was significantly improved (*p* < 0.05), and this effect was strongest at 8 μg/μl. In addition, confocal microscopy was used to evaluate cytotoxicity in cells treated with different concentrations of Sa12b. The results indicated that Sa12b could alleviate the proliferation inhibition of NP-MSCs in a severely acidic environment without inducing cytotoxic effects (Figure 2B).

### Sa12b Reduced the Apoptosis Rate of NP-MSCs in a Severe Acidic Environment

**FIGURE 3:**
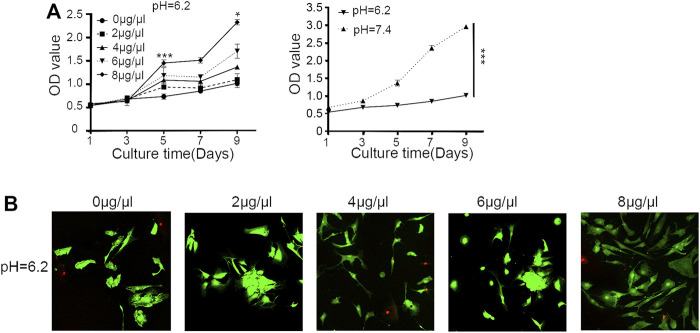
Sa12b alleviated the proliferation inhibition of NP-MSCs in a severely acidic environment. **(A)** The proliferation of NP-MSCs in an acidic environment (*pH* = 6.2) was significantly lower than normal (*pH* = 7.4). When different concentrations of Sa12b (0, 2, 4, 6, and 8 μg/μl) were added, the proliferation inhibition of human degenerated NP-MSCs was significantly improved (*p* < 0.05). *p < 0.05 indicated statistical difference between the groups; ***p < 0.001 indicated significant difference between groups. **(B)** Live and dead cell imaging of NP-MSCs treated with different concentrations of Sa12b. The green fluorescent cells are the live cells labeled with CAM, and the red fluorescent cells are the dead cells labeled with PI.

Human degenerative NP-MSCs were cultured in an acidic environment (*pH* = 6.2) for 2 days, and different concentrations of Sa12b (0, 2, 4, 6, and 8 μg/μl) were added. Flow cytometry was used to evaluate the percentage of apoptotic NP-MSCs after treatment under the above conditions. The results showed that compared with that in the control group, the apoptosis rate of NP-MSCs gradually decreased significantly after adding Sa12b (Figures 3A,B).

### Sa12b Inhibited the Expression of ASICs in the Acidic Microenvironment Conditions and Promoted the Expression of ECM-Related Genes and Proteins

**FIGURE 4:**
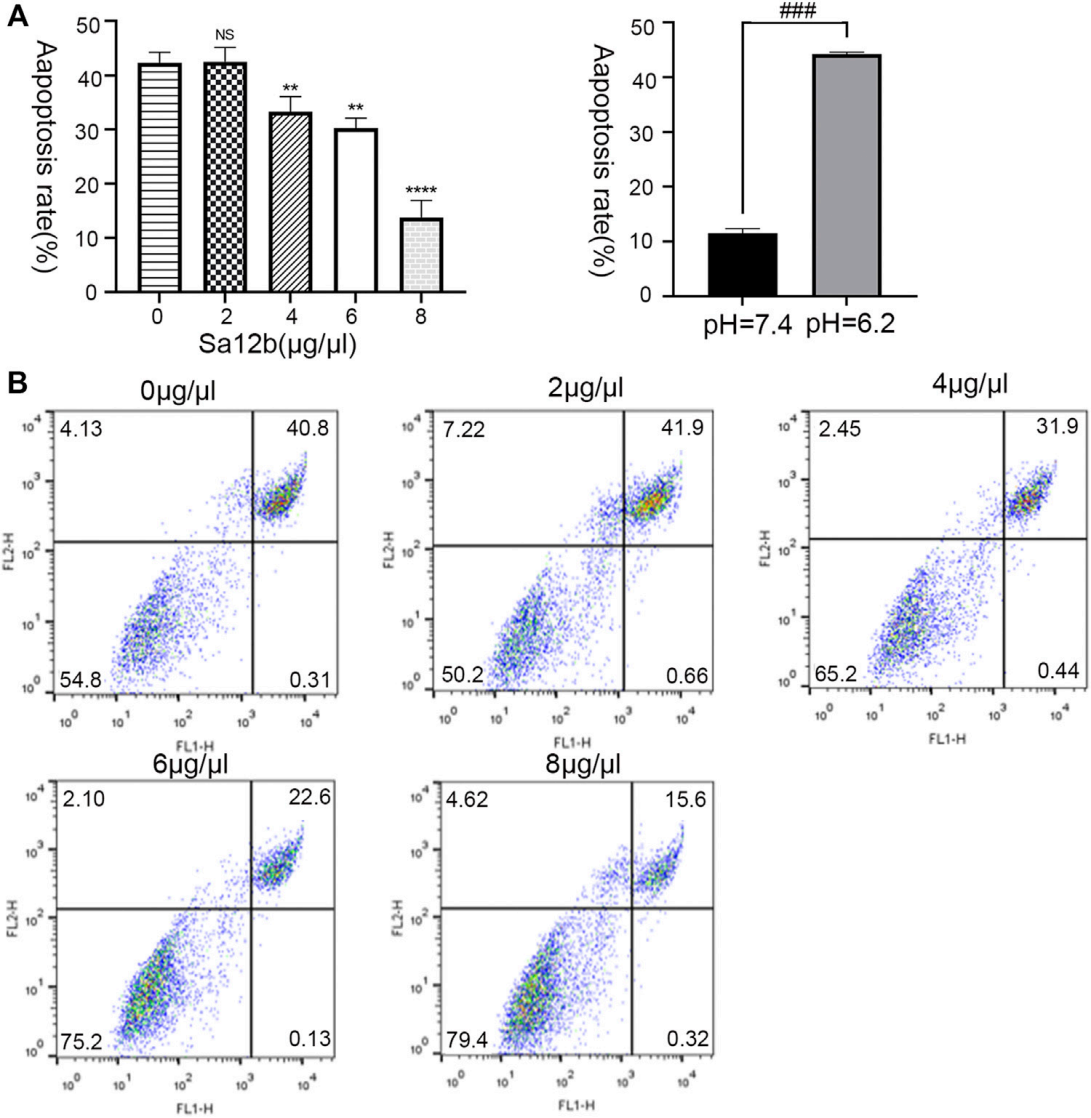
Sa12b reduced the apoptosis rate of NP-MSCs in a severe acidic environment. **(A)** The apoptotic rate of NP-MSCs in an acidic environment (*pH* = 6.2) was significantly higher than normal (*pH* = 7.4). When different concentrations of Sa12b (0, 2, 4, 6, and 8 μg/μl) were added, the apoptotic rate of human degenerated NP-MSCs was significantly reduced. ** indicates *p* < 0.01; **** indicates *p* < 0.0001; NS indicates no statistical significance when the control group (*Sa12b* = 0 μg/μl) was compared with the other groups; ^###^ indicates *p* < 0.001 when the control group (*pH* = 7.4) was compared with the treatment group (*pH* = 6.2). **(B)** Flow cytometry of NP-MSCs treated with different concentrations of Sa12b. The upper right corner indicates the proportion of late apoptosis.

**FIGURE 5:**
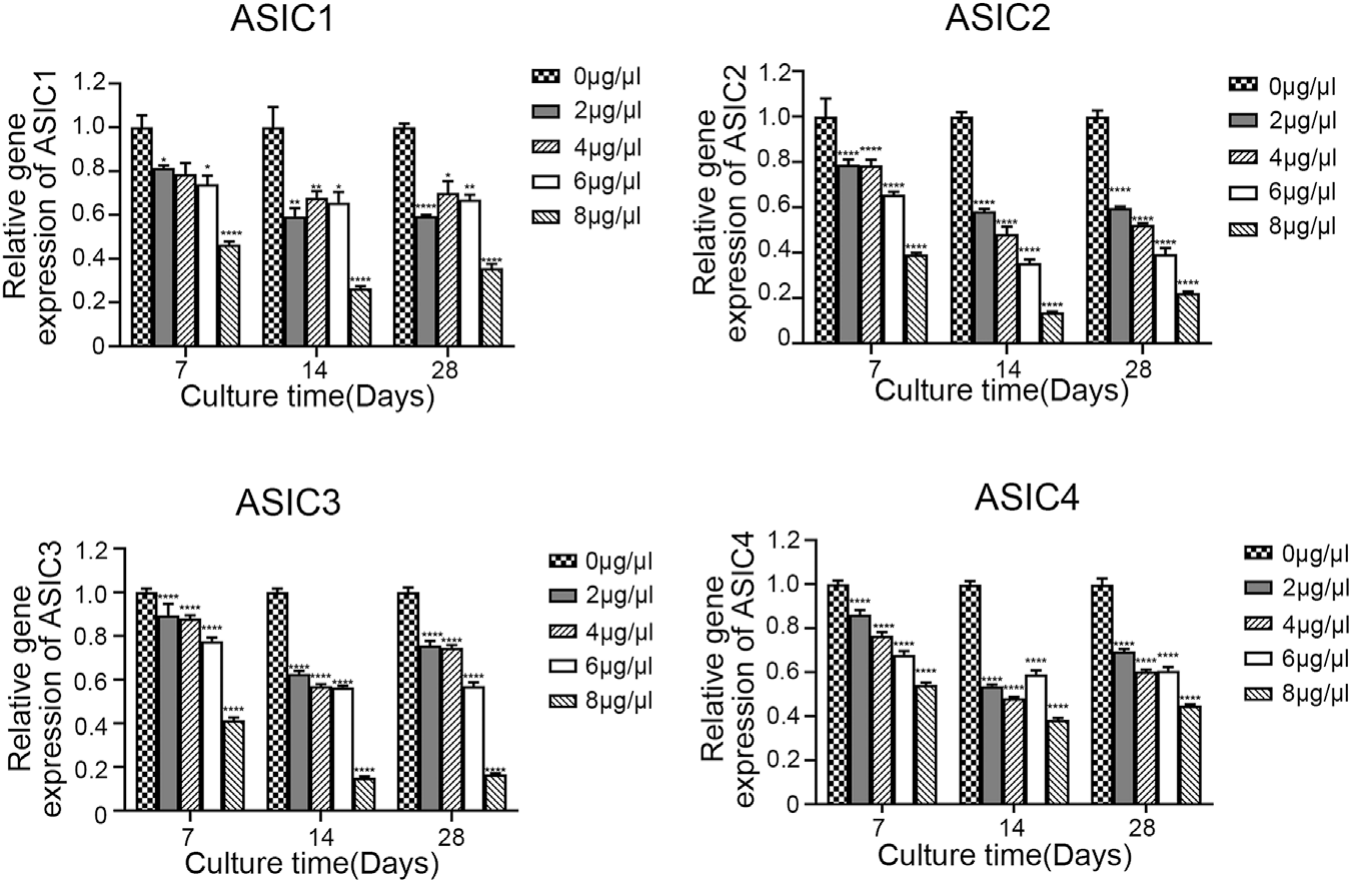
The relative gene expression of ASIC subunits (ASIC1, ASIC2, ASIC3, and ASIC4) gradually decreased with increasing Sa12b concentration. *indicates *p* < 0.05; ** indicates *p* < 0.01; *** indicates *p* < 0.001 when the control group (*Sa12b* = 0 μg/μl) was compared with the other groups.

To evaluate the effect of Sa12b on the subunits of ASICs (ASIC1, ASIC2, ASIC3, and ASIC4) and ECM-related genes (aggrecan, collagen II, and SOX-9) in NP-MSCs in an acidic environment, we used different concentrations of Sa12b (0, 2, 4, 6, and 8 μg/μl). NP-MSCs were cultured for 7, 14, and 28 days. The expression of each gene was detected by qPCR, and GAPDH expression was used as a control. The results showed that, relative to the expression levels of each gene in cells that were not treated with Sa12b, the relative gene expression of the ASIC subunits gradually decreased with the increase in Sa12b concentration (Figure 4), while that of the ECM-related genes gradually increased (*p* < 0.01) (Figure 5). The results also showed that these differences were maintained at least until the 28th day. In addition, western blotting results showed that the expression of ECM-related proteins (aggrecan and collagen II) in NP-MSCs also increased with an increase in Sa12b concentration, which was consistent with the qPCR results.

### Sa12b Increased the Expression of Stem cell-Related Genes Under Acidic Microenvironmental Conditions and Reduced the Influx of Ca^2+^ in NP-MSCs by Inhibiting ASICs

**FIGURE 6:**
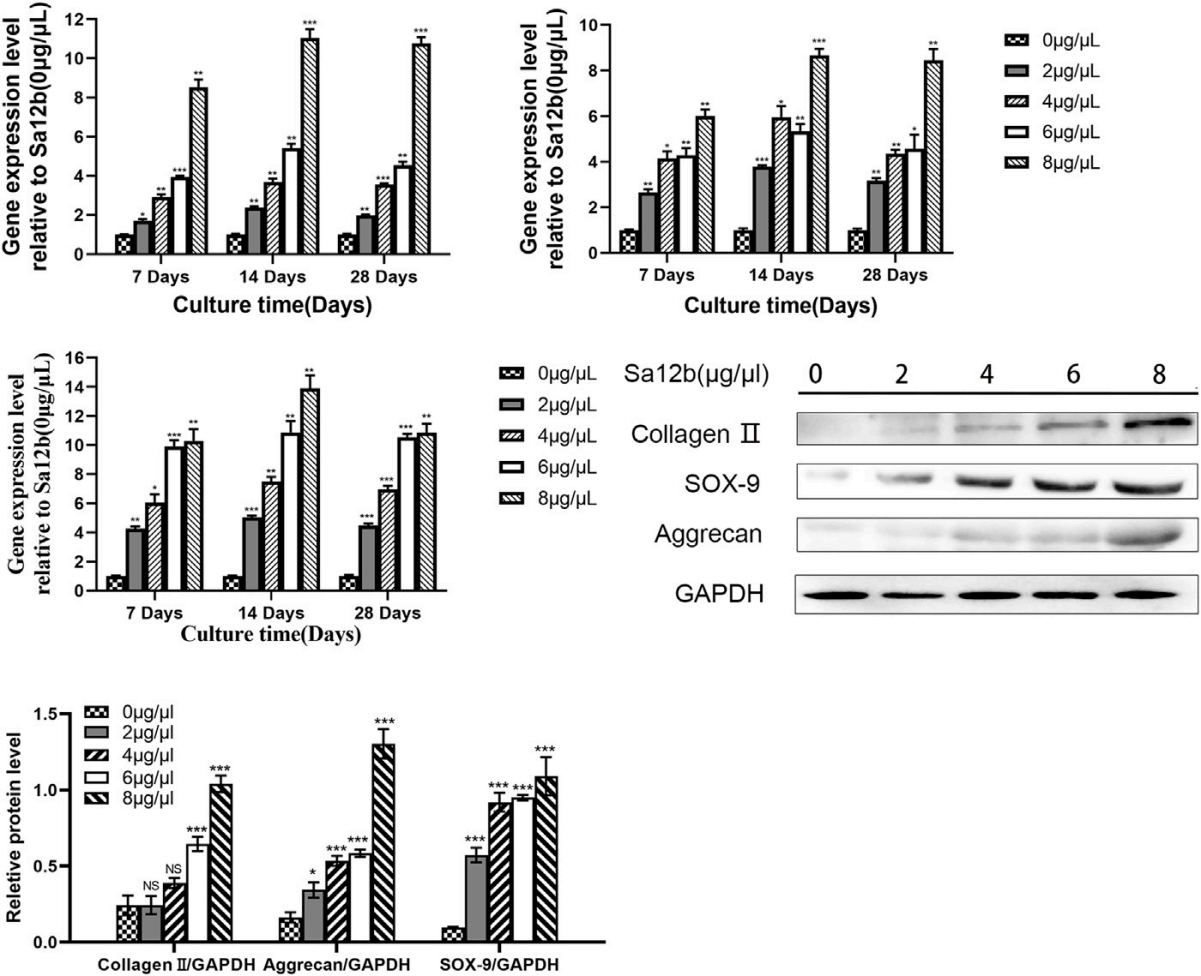
Expression of ECM-related genes (Aggrecan, Collagen II, SOX-9) gradually increased with increasing Sa12b concentration. *indicates *p* < 0.05; **indicates *p* < 0.01; ***indicates *p* < 0.001; NS indicates no statistical significance when the control group (*Sa12b* = 0 μg/μl) was compared with the other groups.

To explore the mechanism by which Sa12b improves the biological activity of NP-MSCs cultured in an acidic environment, we used cells without Sa12b treatment as a control and obtained the optimal Sa12b concentration (8 μg/μl) in the treatment group based on the results of proliferation and apoptosis experiments. As shown in Figure 6, the qPCR results showed that the expression of stem cell–related genes (Oct4, Nanog, Jagged, and Notch1) in the treatment group supplemented with Sa12b was significantly higher than that in the control group (*p* < 0.05) (Figure 6A). To measure Ca^2+^ concentration, we treated NP-MSCs cultured in an acidic environment with the following: no Sa12b, Sa12b at an optimal concentration, amiloride (an ASIC non-specific inhibitor), and Sa12b and amiloride together. The results showed that the Ca^2+^ concentration in the three treatment groups was lower than that in the control group. Interestingly, the Ca^2+^ concentration in the group treated with Sa12b and amiloride simultaneously was significantly less than that in the control group (Figures 6B–D).

## Discussion

During the process of IDD, the change in the microenvironment, especially the low pH, is one of the key factors that inhibit the differentiation of NP-MSCs into NP-like cells (Kanichai et al., 2008; Wuertz et al., 2008; Wuertz et al., 2009; Li et al., 2013; Colombier et al., 2014), mainly by activating ASICs (Liu et al., 2017). In our study, we found that the newly emerged wasp peptide Sa12b (EDVDHVFLRF-), a non-specific ASIC inhibitor, could effectively improve the biological activity of human degenerated NP-MSCs in harsh acidic environments (*pH* = 6.2). Compared with that in the control, the proliferation rate of NP-MSCs increased with the increase in Sa12b concentration, and the apoptosis rate decreased. Moreover, the relative expression of genes encoding ASIC subunits (ASIC1, ASIC2, ASIC3, and ASIC4) expressed in human degenerated NP-MSCs affected by Sa12b was reduced. On the contrary, the expression of genes related to the ECM (collagen II, aggrecan, and SOX-9) and the protein expression of collagen II and aggrecan increased. Therefore, Sa12b improved the biological activity of human degenerated NP-MSCs and is very likely to contribute to remodeling the ECM of the NP tissue and delaying IDD. Furthermore, Sa12b inhibits Ca^2+^ influx in human degenerated NP-MSCs by mediating ASICs to affect proliferation, differentiation, and apoptosis. In summary, our study indicated that the new ASIC non-specific inhibitor Sa12b could improve the biological activity of human degenerative NP-MSCs in a harsh acidic microenvironment and play a beneficial role in the process of IDD.

The chronic decrease in the number and function of NPCs in NP under conditions of IDD and loss of ECM components, especially collagen II and aggrecan, is one of the pathological features of IDD (Antoniou et al., 1996; Roberts et al., 2006; Nicholas and George, 2011; Colombier et al., 2014; Risbud and Shapiro, 2014). Studies have found that NP tissue contains MSC-like cells that can be isolated and proliferated *in vitro*, namely NP-MSCs, which have similar biological activity to bone marrow mesenchymal stem cells (BMSCs) and adipose-derived stem cells (ADSCs) (Blanco et al., 2010; Shen et al., 2015; Clouet et al., 2019), They can self-renew and differentiate into NPCs, promoting the regeneration of ECM after activation (Sakai et al., 2012). NP-MSCs were isolated from the NP in patients who underwent lumbar disc herniation surgery and then were extracted and purified. The homology observed *in vivo* and *in vitro* made this research highly relevant to clinical applications. In the cell identification stage, the cells presented the following characteristics: 1) adherent and long-term growth; 2) high expression of CD73, CD90, and CD105, and low expression of CD34, CD45, and HLA-DR; 3) osteogenic, adipogenic, and chondrogenic differentiation; and 4) expression of genes related to stem cells (Oct4, Nanog, Jagged, and Notch1), which met the international standards of MSCs and is consistent with the research of Shen et al. (Dominici et al., 2006; Shen et al., 2015).

An acidic environment is vital for the progression of IVDD. ASICs, which are closely related to the acidic environment, affect the biological activity of cells by regulating the flow of ions inside and outside the cell (Zhou et al., 2016). Relevant studies have shown that acidic conditions can significantly inhibit the proliferation of human degenerated NP-MSCs and increase cell apoptosis through ASICs. As the pH of the culture environment of human degenerated NP-MSCs decreases, the expression of ASIC1, ASIC2, ASIC3, and ASIC4 gradually increases (Liu et al., 2017). In this study, we found that when the non-specific ASIC inhibitor Sa12b was not added to the culture, the cell proliferation rate decreased as the pH value decreased, and apoptosis increased, which was most apparent when the pH was 6.2 and is consistent with the study by Liu et al. After increasing Sa12b concentration, the proliferation increased, and the apoptosis rate decreased significantly, reaching the optimum level when the Sa12b concentration was 8 μg/μL qPCR showed that when the pH of the complete medium was 6.2 and different doses of Sa12b were added, the relative expression levels of ASIC1, ASIC2, ASIC3, and ASIC4 in NP-MSCs were all reduced. This illustrated that Sa12b affected human degenerative NP-MSCs through ASICs. In addition, studies have shown that endogenous stem cells such as NP-MSCs can self-renew and differentiate into NPCs, which can promote the regeneration of ECM after activation (Sakai et al., 2012). ECM receptor interaction can also activate the AKT pathway to promote the differentiation of neuronal stem cells (He et al., 2021). In addition, liver-specific ECM (L-ECM) can promote the liver differentiation of BM-MSCs by activating specific types of integrins (ITG) and its downstream signal ITG pathways, and play a therapeutically beneficial effect on the liver regeneration of stem cells (Bi et al., 2017). It is consistent with our results, as the concentration of Sa12b increased, the relative expression levels of ECM-related genes and proteins in NP-MSCs, such as collagen II, aggrecan, and SOX-9, were all increased; thus, we speculated that Sa12b had a high probability of promoting the regeneration of ECM in IVDD, promote the differentiation of NP-MSCs through downstream AKT and ITG, and delay IVDD.

In addition, the Notch signaling pathway consists of four Notch receptors (Notch 1–4) and five Notch ligands (Jag1, Jag2, DLL-1, DLL-3, and DLL-4) (Siebel and Lendahl, 2017). The Notch signaling pathway, a key regulator of chondrogenesis, can regulate chondrocyte proliferation and differentiation and maintain the stromal metabolic balance of cartilage in articular chondrocytes (Liu et al., 2016). In addition, the study found that the binding of Notch1 and Jag1 activates the signal molecule HES1, and the activated HES1 inhibits the expression of downstream related genes, thereby inhibiting cell apoptosis and promoting cell cycle progression (Xue et al., 2014; Lee et al., 2016). This study found that the expression levels of Notch1 and Jag1 increased significantly when the Sa12b concentration was optimal (8 μg/μl). Moreover, the qPCR results indicated that the expression levels of each ASICs subunit decreased. Therefore, we believe that Sa12b may activate the Notch signaling pathway by mediating ASICs, increasing the expression of downstream genes in the pathway, promoting proliferation, and inhibiting the apoptosis of NP-MSCs.

Oct4 and Nanog are homologous domain transcription factors that are the core transcription factors of human stem cells. Located upstream of genes responsible for totipotency regulation, Oct4 can maintain the pluripotency of stem cells (Saunders et al., 2017). Oct4 can be directly bound to the Nanog promoter to maintain the activation of Nanog and improve the proliferation ability of cells. Studies have found that upregulation of Oct4 and Nanog expression in dental pulp cells can promote cell proliferation, whereas downregulation can inhibit cell proliferation (Huang et al., 2014). In this study, the expression of Oct4 and Nanog was detected by qPCR, and the results showed that the expression was significantly increased at the optimal Sa12b concentration (8 μg/μl) compared with that in the control group. Therefore, Sa12b may promote the expression of Oct4 and then activate Nanog expression to enhance the pluripotency, proliferation, and differentiation of NP-MSCs.

ASIC1 and ASIC3 are crucial subunits in the cytomembrane and are key mediators of human NP-MSC aging during IVDD. They are affected by changes in the levels of extracellular lactate and induce Ca^2+^ influx. Ca^2+^, a key second messenger in the signal transduction pathway, induces the activation of the NF-κB signaling pathway, leading to apoptosis (Ding et al., 2021; Zhao et al., 2021). *In vitro* animal experiments have also shown that ASIC1 mediates cartilage endplate apoptosis and matrix metabolism under acidic conditions (Yuan et al., 2016). In addition, the reduction in the pH of the articular fluid can lead to excessive apoptosis of chondrocytes. Wu et al. found that ASIC1a may promote [Ca^2+^] i and upregulate NLRP3 inflammasome expression, thus mediating cocaine death in AA rat chondrocytes (Wu et al., 2019).

Our results showed that the Ca^2+^ influx in the three treatment groups was lower than that in the control group. Interestingly, the Ca^2+^ influx in the group treated with Sa12b and amiloride simultaneously was significantly less than that in the control group. Studies have shown that in articular cartilage tissue and IVD, when the pH is within the physiological range, Ca^2+^ has a very high affinity for ASICs and can bind to the outside of the channel holes to close the channels; when pH decreases, H+ binds to acid-sensitive sites on the ASICs, reducing the binding affinity of Ca^2+^ to the ASICs, and Ca^2+^ leaves the channel pore, promoting the opening of the ASICs and entering the cell (Zhang et al., 2020). In addition, scientists speculated that the inhibitory effect of Sa12b on ASICs may occur because Sa12b has two positively charged residues (His5 and Arg9) that can bind to the binding site of the ASIC, especially the central vestibule of the channel, thereby blocking the channel (Hernández et al., 2019). Moreover, related studies have shown that ASIC1 and ASIC3 activate the senescence programming pathway of P53-P21/P27 and P16-RB1 signaling in an acid environment, and the expression of genes P53, P21, P27, P16 and RB1 increases, which promotes human degenerative disc NP- MSCs senescence and degeneration (Ding et al., 2021). The acid environment also regulates the level of reactive oxygen species between NP cells differentiated from human degenerated intervertebral disc NP-MSCs through ASIC1 and ASIC3, activates the NF-κB signaling pathway, and promotes intracellular activation of the NLRP3 inflammation group and IL-1β release, thereby promoting NP degeneration (Zhao et al., 2021). Therefore, we believe that Sa12b is very likely to reduce the Ca^2+^ influx by inhibiting AISCs, reduce the expression of genes related to the aging programming pathway of P53-P21/P27 and P16-RB1 signal transduction, and improve the biological activity of human degenerated intervertebral disc NP-MSCs. And inhibit or avoid activation of NF-κB signal to reduce the expression of inflammatory factors, thereby delaying IVDD.

Our study demonstrated that in the harsh acidic environment (*pH* = 6.2), the inhibition of AISCs reduced the influx of Ca^2+^, thus improving the biological activities of NP-MSCs, such as proliferation, differentiation, and apoptosis. However, there were still some limitations in our study. First, we only studied the influence of Sa12b on the biological activity of NP-MSCs in a harsh acid environment, so further research on other IVD environments at different pH levels should be conducted. Second, although Sa12b reduces Ca^2+^ influx by inhibiting AISCs, thereby mediating the improvement of the biological activity of NP-MSCs, the specific signal pathways through which Sa12b affects NP-MSCs need further study. Third, we only carried out *in vitro* studies on the biological activity of NP-MSCs in the model of an acidic environment. We did not investigate the effects of various factors *in vivo*. Therefore, animal experiments should be conducted in future studies.

In summary, our study indicated that Sa12b reduces Ca^2+^ influx by inhibiting AISCs in the harsh acidic environment of IVD and may improve the biological activity of NP-MSCs through the Notch signaling pathway. This study provides a new perspective for the biological treatment of IVDD. Sa12b has enormous therapeutic potential for delaying IVDD and improving low back pain.

**FIGURE 7:**
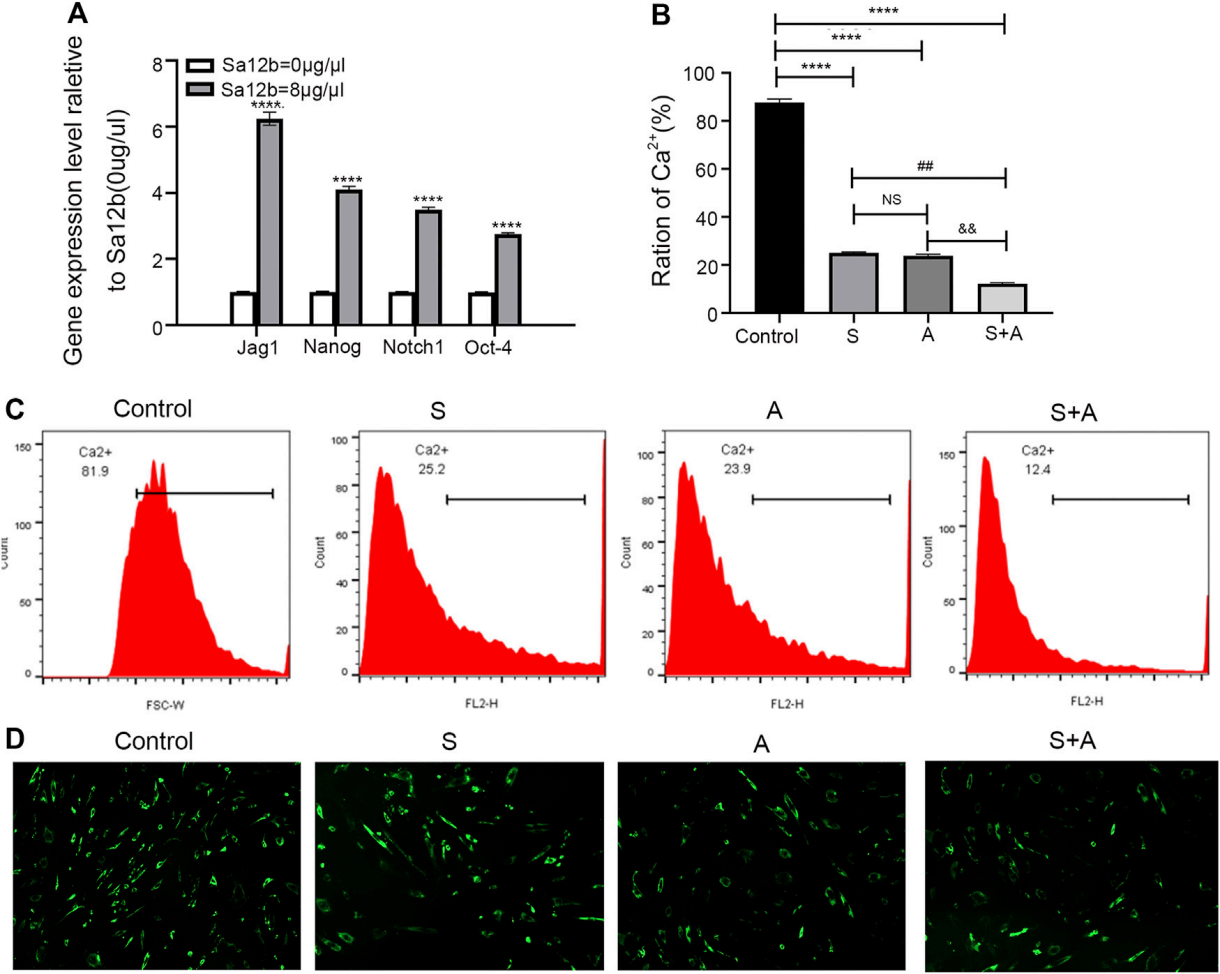
Sa12b increased the expression of stem cell–related genes under acidic microenvironmental conditions and reduced the influx of calcium ions in NP-MSCs by inhibiting ASICs. **(A)** In an acidic environment, the optimal concentration of Sa12b was applied to NP-MSCs, and the expression of stem cell–related genes (Jag1, Nanog, Notch1, and Oct4) was significantly higher than that in the control group (*p* < 0.001). ****indicates *p* < 0.0001 when the control group (*Sa12b* = 0 μg/μl) was compared with the other groups; NS indicates no statistical significance when the Sa12b treatment compared with the group with amiloride. ^*##*^indicates *p* < 0.01 when the Sa12b treatment (*Sa12b* = 8 μg/μl) compared with the group with amiloride and Sa12b. ^&&^indicates *p* < 0.01 when the Sa12b group compared with the amiloride and Sa12b treatment. **(B)** The Ca^2+^ concentration in the treatment groups (Sa12b, amiloride, and combined Sa12b/amiloride) was lower than that in the control group according to the results of flow cytometry. Furthermore, the Ca^2+^ concentration in the group administered Sa12b and amiloride simultaneously was significantly lower than that in the control group. **(C)** Ca^2+^ concentration was measured by flow cytometry. **(D)** Confocal laser scanning microscopy imaging analysis of Ca^2+^ concentration. *p < 0.05, **p < 0.01, ***p < 0.001.

## Data Availability

The original contributions presented in the study are included in the article/Supplementary Material, further inquiries can be directed to the corresponding authors.
